# The power of community science to quantify ecological interactions in cities

**DOI:** 10.1038/s41598-021-82491-y

**Published:** 2021-02-04

**Authors:** Breanna J. Putman, Riley Williams, Enjie Li, Gregory B. Pauly

**Affiliations:** 1 Urban Nature Research Center, The Natural History Museum of Los Angeles County, 900 Exposition Boulevard, Los Angeles, CA 90007 USA; 2 Present Address: Department of Biology, California State University, San Bernardino, 5500 University Parkway, San Bernardino, CA 92407 USA

**Keywords:** Behavioural ecology, Urban ecology

## Abstract

Studying animals in urban environments is especially challenging because much of the area is private property not easily accessible to professional scientists. In addition, collecting data on animals that are cryptic, secretive, or rare is also challenging due to the time and resources needed to amass an adequate dataset. Here, we show that community science can be a powerful tool to overcome these challenges. We used observations submitted to the community science platform iNaturalist to assess predation and parasitism across urbanization gradients in a secretive, ‘hard-to-study’ species, the Southern Alligator Lizard (*Elgaria multicarinata*). From photographs, we quantified predation risk by assessing tail injuries and quantified parasitism by counting tick loads on lizards. We found that tail injuries increased with age and with urbanization, suggesting that urban areas are risky habitats. Conversely, parasitism decreased with urbanization likely due to a loss of hosts and anti-tick medications used on human companion animals. This community science approach generated a large dataset on a secretive species rapidly and at an immense spatial scale that facilitated quantitative measures of urbanization (e.g. percent impervious surface cover) as opposed to qualitative measures (e.g. urban vs. rural). We therefore demonstrate that community science can help resolve ecological questions that otherwise would be difficult to address.

## Introduction

As the human population continues to grow and become more urban^1^, animals increasingly have to survive and reproduce in modified habitats. Human-induced habitat modifications, like urbanization, shift ecological relationships including inter- and intra-specific competition and predator–prey and host-parasite interactions^2–4^. Of specific interest are shifts in predation and parasitism as these have direct and indirect effects on species’ life history traits^5–7^. If such relationships are altered so much that animals lack the phenotypic plasticity or evolutionary potential to respond, this could eliminate animals from urban habitats^8^. Although shifts in ecological relationships are likely to occur with increasing urbanization, there remain several barriers to studying them. First, much of the urban landscape is private property, and gaining access to conduct research can be logistically challenging if not impossible for broader studies. Second, and not unrelated to the first point, studying the ecology of organisms that are secretive or rare is logistically difficult because of the large temporal and/or spatial scales required to collect an adequate dataset.

Community science (also called citizen science), which involves scientists partnering with members of the public to answer research questions, has the potential to fill these data gaps in urban ecology research. Platforms such as Zooniverse (https://www.zooniverse.org/), eBird (https://ebird.org/), and iNaturalist (https://www.inaturalist.org/) collate millions of observations of thousands of species worldwide. These large datasets of species occurrence records across large geographic scales have been used successfully to model species distributions, abundances, and overlap^9^, to evaluate changes in phenology^10^, and for biodiversity assessments^11^. Yet, most ecological studies using community science-generated species occurrence records narrowly use the spatial (i.e. latitude and longitude) and/or temporal (i.e. date and time) data associated with the observations. We expand on the uses of community science-generated data by demonstrating that the content of submitted images contains valuable untapped data. From submitted photographs, we quantify ecological interactions that affect animal fitness—predation and parasitism. Because the spatial extent of these observations covers the entire spectrum of urbanization, we could examine how these two key aspects of a species’ ecology change over a human-modified landscape.

There is conflicting evidence on the directions in which urbanization affects predation and parasitism. Predation could be lower in urban areas because of the human shield effect^3,12^ whereby predators are repelled by human presence, leading to relatively safe habitats for prey^13^. Many urban animals, however, suffer serious predation by human companion animals such as owned or feral cats and dogs^14–16^. As for parasitism, animals living in urban areas often have poorer body conditions^17,18^, are exposed to higher levels of environmental contaminants^19^, and can experience higher population densities^20^, all of which could enhance parasitic infections^21^. Indeed, some urban populations of lizards^22^, birds^23^ and rodents^24^ have higher levels of parasitic infections compared to non-urban populations. Nonetheless, the abundance and diversity of appropriate hosts/vectors are often reduced in urban environments, effectively lowering levels of parasitism in urban animal populations^25^.

Here, we use photo-vouchered community science observations posted to the Reptiles and Amphibians of Southern California (RASCals) project (https://www.inaturalist.org/projects/rascals) to assess how predation and parasitism scale with urbanization in a widespread, but secretive species, the Southern Alligator Lizard (*Elgaria multicarinata*). RASCals is hosted on iNaturalist and incorporates observations of herpetofauna from Southern California. Since its inception in 2013, over 58,000 observations have been added. Southern Alligator Lizards are the second most common species posted to RASCals, with over 6700 observations as of December 2020. We recognize that many community science platforms exist, and we chose iNaturalist because it is taxonomically diverse (as opposed to eBird), used worldwide (notably it is the planform of choice for the global City Nature Challenge event; https://citynaturechallenge.org), and data undergo quality assessments and are easily downloadable for use. Therefore, the methods we use in this study could be implemented by researchers around the world and would apply to other species or taxonomic groups. We chose Southern Alligator Lizards as our focal species because they are infrequently observed relative to similarly distributed diurnal lizards and therefore difficult to study using traditional field methods. They are a solitary, secretive species found in both natural and urban habitats in the western United States and Mexico, and they generally avoid basking, preferring cooler temperatures and spending their time under logs, rocks, surface cover, vegetation, and other dark, moist microhabitats. Even experienced field ecologists are unlikely to see more than one a day in urban habitats and more than a handful per day in non-urban areas. Thus, there are few published studies on the ecology of this species. However, as we demonstrate, when a species has low encounter rates (i.e. a single observer may only encounter 1–2 lizards during a one-day survey), community science can generate numerous observations by increasing the number of observers looking for lizards (whether intentionally or not).

We measured predation risk by quantifying tail breaks in lizards from community science-generated photographs. Southern Alligator Lizards have long, semi-prehensile tails which they readily autotomize (self-amputate) as an escape tactic against predators. Tail autotomy is thought to be an indicator of predation intensity^26,27^; it can also occur through intraspecific aggression^28^, but intraspecific aggression is not documented as resulting in tail autotomy in alligator lizards^29^. The loss of the tail is not insignificant and has been shown to have serious fitness costs, including decreasing locomotor performance, increasing susceptibility to predation, lowering social status, increasing metabolism to replace lost tissue, and decreasing fecundity^27,30^. Thus, if predation risk increases with intensity of urbanization, then lizards living in the most-urban environments might experience these fitness costs associated with tail loss. Although several studies indicate animals have lower predation risk in urban areas, domestic or feral cats are common and kill an estimated 258–822 million reptiles each year in the United States^14^. One study found that urban anole lizards in Puerto Rico had significantly more tail breaks than anoles living in natural areas, suggesting urban habitats are riskier than natural ones, but this is the only study of this kind^31^. Based on this previous literature, iNaturalist observations of cats injuring and/or killing lizards (Supplementary Fig. 1), and our own experiences with cats and lizards, we predicted that the probability of tail breaks would increase with urbanization.

**Figure 1:**
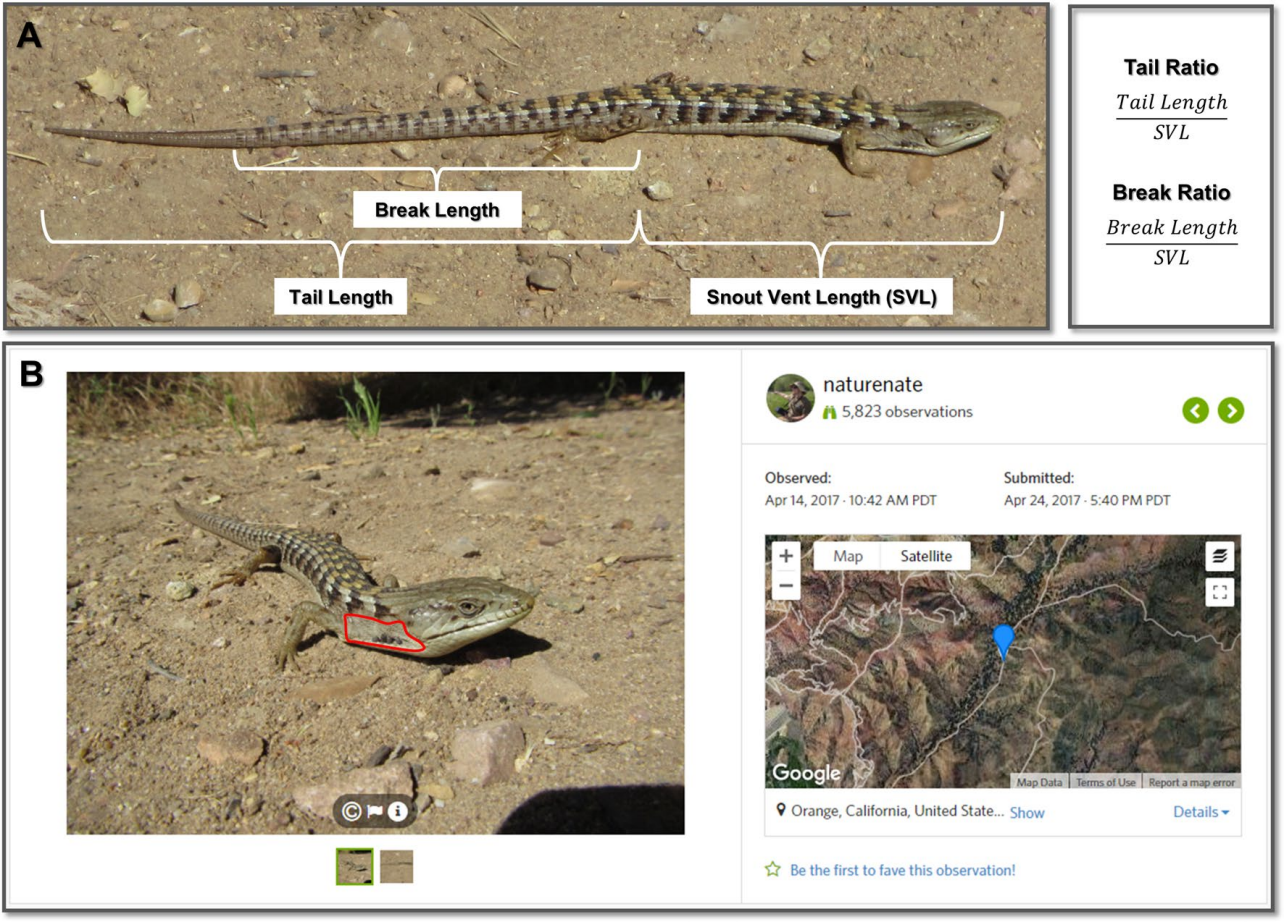
An observation of a Southern Alligator Lizard submitted to iNaturalist with two photographs, both of which were analyzed, showing the traits quantified in this study. (**A**) Body and tail measurements quantified to estimate predation risk. (**B**) The ear region (outlined in red) from which we estimated tick parasitism (with ticks present). The righthand side of the observation shows other data that were extracted including the date and time of observation, the date and time of submission, and the geographic locality. Photo and observation by Nathan Smith (iNaturalist user naturenate; iNaturalist 5945401; https://www.inaturalist.org/observations/5945401). Map data: 2021 Google, 2021 INEGI Imagery and 2021 TerraMetrics.

In addition, we examined whether the tail ratio (relative length of unbroken tails) and break ratio (relative length of the original tail for lizards with tail breaks) vary with urbanization (Fig. 1A). Tail autotomy in lizards typically occurs in fracture planes within individual vertebra. Regrown tails lack vertebrae and instead have a stiff cartilaginous rod for support; thus, subsequent autotomy events tend to occur anterior to the prior break site^32,33^. If the break ratio associates negatively with urbanization, this would suggest that tail breaks are occurring more frequently within individual lizards in urban environments^34^. Variation in tail ratios along an urban gradient could indicate shifts toward altered morphologies due to selection, phenotypic plasticity, and/or spatial sorting.

We measured parasitism by quantifying the prevalence and intensity of California Black-legged Tick (*Ixodes pacificus*) infection in lizards from community science observations (Fig. 1B). From photographs, we noted whether any ticks were present (ectoparasite prevalence) within the ear region and if so, we counted the number present (intensity of infection). Southern Alligator Lizards are a primary host of subadult ticks^35^. Because the final hosts for adults of this tick species are large mammals, which are generally lower in abundance in urban areas (e.g. deer) or are treated with anti-tick medications (e.g. dogs and cats), we expected to see the prevalence and intensity of ectoparasite infections decrease with urbanization.

## Results

By extracting data from a two-year period, we achieved a relatively large dataset of 1688 Research Grade observations of Southern Alligator Lizards uploaded by 424 community scientists to the RASCals iNaturalist project. Of the 1688 observations, 723 met our criteria (see “Methods”) to be included in the predation study (from 386 unique observers; 1.71 ± 3.84 observations per observer [mean ± SD]; Supplementary Fig. 2A) and 157 met the criteria to be included in the parasitism study (from 114 unique observers; 1.38 ± 0.96 observations per observer [mean ± SD]; Supplementary Fig. 2B). Importantly, the observations spanned a broad geographic range (Supplementary Fig. 3) allowing us to quantify a continuous measure of urbanization intensity—percent impervious surface cover. This alligator lizard research was not advertised; we simply harvested observations from the RASCals project that fit the criteria. Observations were reviewed and selected for inclusion over a short time period (approximately 3 weeks) as time allowed, highlighting that community science platforms house opportunities for research beyond the use of the typical spatial and temporal data.

### Effect of urbanization on predation

**Figure 2:**
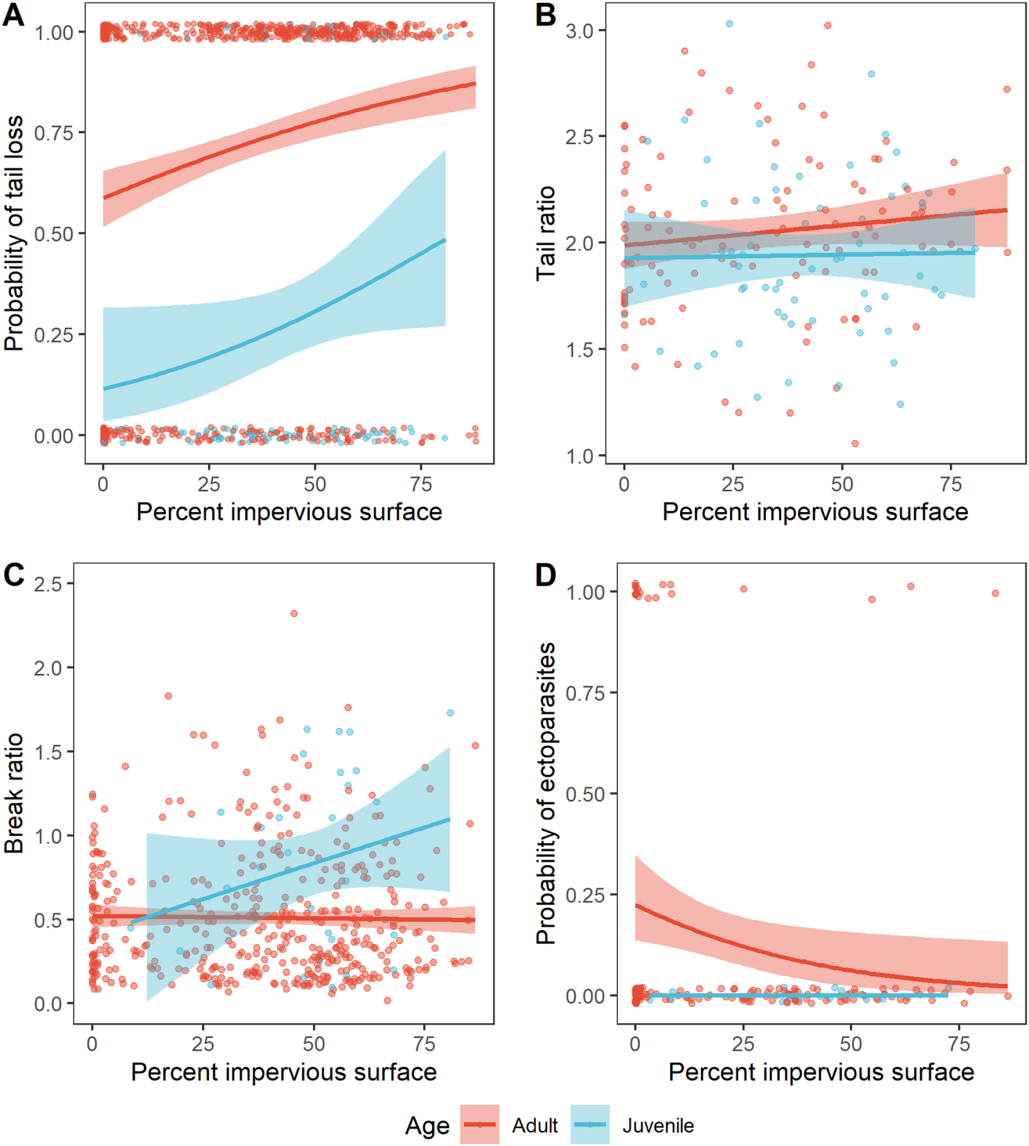
Effects of age and urbanization intensity on predation and parasitism in Southern Alligator Lizards. (**A**) Probability of tail loss (yes = 1, no = 0) (N = 723); (**B**) Tail ratio for lizards with unbroken tails (N = 171); (**C**) Break ratio for lizards that had experienced tail loss events (N = 455); (**D**) Probability of ectoparasitic infections (yes = 1, no = 0) (N = 157). Lines and standard errors generated from logistic regression models (**A**,**D**) or general linear models (**B**,**C**). For simplicity, the standard error has been removed from the “Juvenile” line in (**D**) because with no infected juveniles, the standard error is 1.

The frequency of tail breaks increased with urbanization (N = 723, Estimate = 0.018, SE = 0.004, Z = 5.036, P < 0.001, Fig. 2A). For every 10% increase in impervious surface cover, the probability of tail loss increased by an average of 2.65 percentage points. In high density residential neighborhoods, the probability of tail loss was more than 20 percentage points higher than in a completely natural habitat (i.e. 0% impervious surface). There was no effect of urbanization on the relative length of unbroken tails (i.e. tail ratio; N = 171, X^2^ = 1.451, df = 1, P = 0.228, Fig. 2B), but adults tended to have longer, unbroken tails in relation to their body length compared to juveniles (mean ± SD adults: 2.05 ± 0.38, N = 109; juveniles: 1.94 ± 0.38, N = 62; X^2^ = 3.746, df = 1, P = 0.053). This suggests positive allometric scaling of the tail occurs in this species. For alligator lizards that had suffered tail loss events, we did not find an effect of urbanization on the relative length of the original tail remaining (i.e. break ratio; N = 455, X^2^ = 0.599, df = 1, P = 0.439, Fig. 2C). Juvenile lizards with tail breaks, however, had significantly more of the original tail left in relation to their body size compared to adults with tail breaks (mean ± SD adults: 0.51 ± 0.39, N = 425; juveniles: 0.83 ± 0.54, N = 30; X^2^ = 10.81, df = 1, P = 0.001). Juveniles were also less likely to have tail breaks than adults (adults N = 618; juveniles N = 105; Estimate = − 2.085, SE = 0.244, Z = − 8.548, P < 0.001; Fig. 2A). These two results corroborate past work which has shown that tail breaks accumulate with age^30^.

### Effect of urbanization on parasitism

Ectoparasite prevalence decreased with urbanization (N = 157, Estimate = − 0.029, SE = 0.013, Z = − 2.25, P = 0.024, Fig. 2D). In urban habitats with 80% impervious surface cover, the probability of having ectoparasites was 2%, while this probability reached more than 20% in completely natural habitats (i.e. 0% impervious surface). It is notable that most infected lizards occurred in areas with less than 20% impervious surface cover (Supplementary Table 1), the cutoff for land classified as open space in the National Land Cover Database. Fifty percent of lizards with ticks in open space land had 3 or more ticks (maximum of 5), whereas all lizards found in more developed land classes had only one or two ticks (Supplementary Table 1). Age had no effect, although we did not find any ticks on juveniles in the dataset (Estimate = − 16.34, SE = 1642.83, Z = − 0.010, P = 0.992).

## Discussion

Collectively, we demonstrate that community science can be a powerful tool to answer modern ecological questions. Large datasets that span broad spatial scales were easily obtained in a relatively short time period. We collected more data on a secretive species and at a faster pace than would be possible using traditional field methods. This type of study would have been limited without the use of community science data because (1) the focal species is secretive and has low detectability through traditional field surveys, (2) most urban sites in Southern California and elsewhere are private property and therefore not easily accessible, and (3) the costs associated with the time, personnel, and money needed to perform adequate data collection across a gradient of urbanization intensities would be relatively high.

Our results refute the idea that urban animals experience a human shield (i.e., humans shield them from natural predators). Adult lizards living in the most urbanized areas have a 75–80% probability of having lost their tails, which is more than a 20-percentage point increase compared to adults living in the most natural sites. It is likely that for small-bodied, dispersal-limited organisms, such as most herpetofauna, cats are a major source of mortality in urban habitats (see Ref.^14^). Tail breaks may also increase with urbanization because of a higher likelihood of encountering other risks such as vehicles, bicycles, people, and other human-subsidized predator populations. In support of this, one study found that Galapagos lava lizards (*Microlophus albemarlensis*) living near roads had a higher frequency of tail breaks than those farther from roads^36^. Regardless, lizards living in urban environments likely experience the fitness costs associated with tail breaks.

We did not find an effect of urbanization on the tail break ratio, the relative length of the original tail remaining for lizards that had experienced a tail break. The break ratio is an indirect measure for the number of tail loss events experienced by individual lizards as tail breaks are most likely to occur along vertebrae in the original tail. Our result that adults had lower break ratios than juveniles, suggests that lizards experience multiple tail breaks throughout their lives, corroborating past research.

As predicted, we found that ectoparasite prevalence decreased with urbanization. This decline is likely due to disruptions in the tick’s life cycle in urban environments. The final hosts for adult female ticks are medium to large mammals such as cervids, canids, and felids. Populations of these taxa are generally lower in abundance in urban areas than in natural ones. Domestic pets such as cats and dogs are also suitable final hosts, but humans often use anti-tick medications to reduce tick attachment on these animals. Thus, it appears that lizards are released from tick parasitism in increasingly urban areas. Urban lizards could therefore experience fitness benefits from reduced parasitism as costs of tick infestations include smaller home ranges, less movement, lower sprint speed, and lower endurance^37^.

Community science has the potential to increase our knowledge of secretive, cryptic, or rare taxa. With low encounter rates, alligator lizards are logistically difficult to study and little is known about basic aspects of their ecology. Studies that use traditional field techniques generally have lower sample sizes, take several years, and require costly methods to conduct. For example, over a 22-year time period, Stanley et al. recorded 582 individual alligator lizards (about 26 lizards per year) at a single urban preserve, but of these, only 27 were juveniles, meaning that researchers were capturing less than two juveniles per year^38^. In our dataset, we were able to obtain observations of 105 juveniles and 618 adults over a 2-year time period and across a gradient of urbanization intensities. Furthermore, this one study had to deploy 18 pitfall trapping arrays just to get this dataset. These traps are costly and must be repeatedly checked and maintained by a dedicated field crew. Even data collection on abundant and conspicuous species is not as efficient as we have demonstrated here. For instance, Tyler et al. collected 55–201 Puerto Rican Crested Anoles (*Anolis cristatellus*) at four sites to evaluate tail loss frequency in urban and rural populations, but these data took three years to collect whereas we downloaded and analyzed over 1600 observations in less than a month. Furthermore, because community science-generated data cover a large geographic extent, we were able to evaluate the impacts of a continuous measure of urbanization on predation and parasitism. This is in contrast to many urban ecological studies that lump populations into urban versus non-urban categories^31,39,40^ despite there being a great deal of variation across urbanized habitats^11^.

In sum, community science platforms can provide more than just spatial and temporal data of species occurrences. There is additional valuable information that can be gleaned from the images uploaded as photo-vouchers to platforms like iNaturalist. iNaturalist is just one of many community science platforms that gather such data and therefore the methods we describe here are not limited to this single source or to a single taxonomic group. We used photo-vouchers to study risk-associated trauma (tail breaks) and ectoparasite attachment (ticks) in a single species, but our approach can be applied broadly to study the ecology of any identifiable taxon in human-modified habitats or in situations where crowdsourcing data could be more effective than using traditional data collection approaches. For many research questions, the pace and scale of data collection through community science far exceeds traditional research methods and can help further our understanding of anthropogenic impacts on wildlife worldwide.

## Methods

Photo-vouchered observations of Southern Alligator Lizards (*Elgaria multicarinata*) were sourced from the Reptiles and Amphibians of Southern California (RASCals) project on iNaturalist (www.inaturalist.org/projects/rascals). We only included Research Grade observations that were submitted during a two-year period: October 2015 to September 2017. Research Grade means that observations have a photo voucher, locality information, date of the observation, and a community-supported taxonomic identification (Fig. 1). The locality information includes latitude and longitude as well as the locational accuracy. We only included observations with accuracy values less than 1 km. For observations less than 100 m apart, we visually compared the size, color, dorsal barring pattern, and tail break presence and length dimensions of lizards in the images to eliminate potential duplicates of the same individual. Of 723 observations, 182 were of two adults observed within 100 m of each other and 16 were of two juveniles observed within 100 m of each other. We also looked at pairs of observations in which a juvenile was observed within 100 m and at least 6 months prior to an adult to account for a juvenile transitioning into an adult during our study’s 2-year timeframe (thus, the same individual could be observed twice). Of the 723 observations, 8 met these criteria. We visually inspected these pairs of observations as above and determined that none were likely to be of the same individual. We quantified the mean percent impervious surface in a 100-m radius of each observation, based on the National Land Cover Database (NLCD)-2016 Percent Developed Imperviousness layer, and used this as a proxy for urbanization intensity.

Observations (N = 723) were used in the predation portion of the study if the entire tail was clearly visible (including unbroken tails, broken tails that had not regrown, and regrown tails; Fig. 1A). We used the Segmented Line tool in ImageJ to measure the following: (1) snout-vent length (SVL)—from the tip of the snout to the location of the vent (cloaca), (2) tail length—from the vent to the posterior tip of the tail, and (3) break length—from the vent to the location of the break, if present (Fig. 1A). When a ventral view of the lizard was not available, which was the case with most observations, the vent was approximated to be 1–2 scale rows posterior to the hind limb insertion; this value was selected following examination of preserved *E. multicarinata* specimens at the Natural History Museum of Los Angeles County. We then calculated the tail ratio (tail length/SVL) for lizards with original unbroken tails and the break ratio (break length/SVL) for lizards that had experienced tail loss events.

Observations (N = 157) were used in the parasitism portion of the study if the entire ear region was visible (Fig. 1B) and individual scales in this region could be detected. This scale criterion was used because if these small scales could be detected, then individual nymphal ticks, which are a similar size, could also be counted if present. We chose to focus on the ear region because a preliminary examination of photographs and museum specimens revealed that this is the most common attachment area for ticks on *E. multicarinata*. We defined the ear region as the area with small scales on the side of the lizard between the ear opening and the forelimb insertion, above the ventral skin fold and below the larger, often keeled, scales of the dorsum (Fig. 1B). We excluded the ear opening as this was not consistently visible. We noted whether any California Black-legged Ticks (*Ixodes pacificus*) were present (ectoparasite prevalence) and if so, counted the number present (intensity of infection).

We categorized each lizard as either juvenile or adult based on dorsal pattern. Southern Alligator Lizards undergo an ontogenetic shift in color pattern with juveniles having a broad brown or tan stripe down their backs with no or minimal transverse dorsal bars; as they age, distinct dorsal bars appear. Juveniles were defined as lizards with incomplete or absent barring, while adults were described as individuals with complete barring (juvenile: *N* = 105; adult: *N* = 618).

From a subset of the observations we identified sex based on mating behaviors because males bite females on the neck, holding this position, before, during, and after mating^29^. Based on submitted observations of mating behavior, we could determine the sex of a subset of individuals (female: *N* = 59, male: N = 75).

We examined the effects of urbanization (percent impervious surface) and age (juvenile or adult) on tail loss and ectoparasite prevalence using multiple logistic regressions. We looked for interactions between impervious surface and age and found none. We assessed whether the tail ratio and break ratio varied with age and urbanization using general linear models. Tail ratio and break ratio were log-transformed prior to analyses. We used observations with known sex (N = 134) to look for sex differences in tail loss, tail ratio, and break ratio and found none (all P > 0.05). We were unable to compare sex differences in ectoparasite presence because of small sample sizes (female: N = 1, male: N = 12). We did not include sex as a factor in further analyses. Furthermore, low sample sizes prevented us from assessing the effect of urbanization on intensity of ectoparasite infection (N = 20 infected individuals, mostly with a single tick). Thus, we report the results from logistic regression on ectoparasite prevalence and descriptive statistics on intensity of infection. Finally, we looked for spatial autocorrelation in the data by testing whether the residuals of our logistic regression model are spatially independent. We did this by calculating Moran’s *I* coefficient and found no significant spatial dependence (Moran's *I* = − 0.014; P = 0.40). All analyses were run in R 3.6.1 (R Core Team, 2017).

## Supplementary Information

Supplementary Information 1.

Supplementary Information 2.

## Data Availability

All data analyzed during this study is included in the published article as a Supplementary Information file.
