# Supplementary material for: The power of community science to quantify ecological interactions in cities
Source: Sci Rep. 2021 Feb 4;11:3069. doi: 10.1038/s41598-021-82491-y (PMC7862361; doi:10.1038/s41598-021-82491-y)
Supplement: Supplementary file 1 — Supplementary Information 1. [file 41598_2021_82491_MOESM1_ESM.docx]

The Power of Community Science to Quantify Ecological Interactions in Cities

Breanna J. Putman, Riley Williams, Enjie Li, Gregory B. Pauly

**Supplementary Figures and Tables**

**
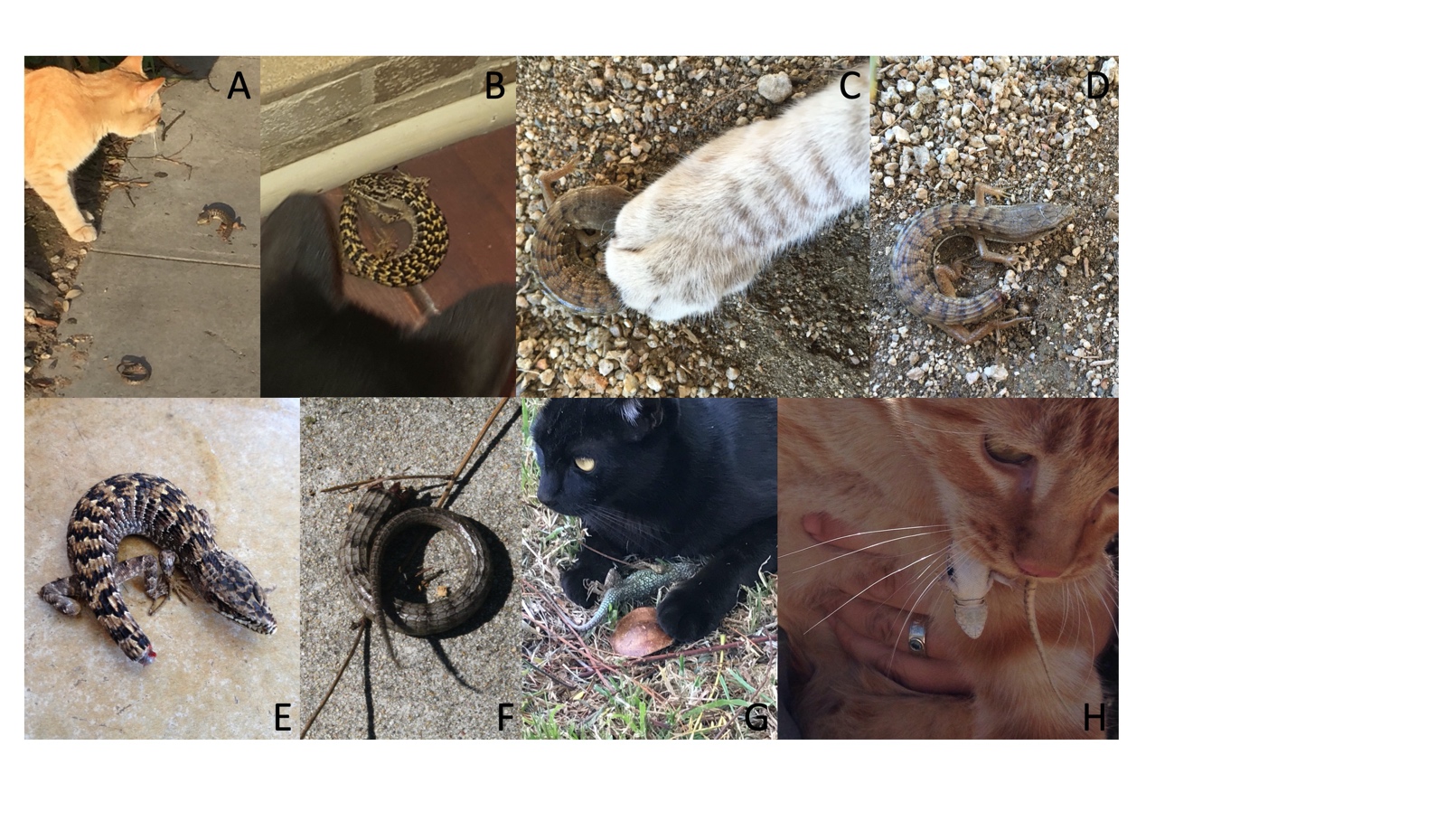
**

**Supplementary Fig. 1.** iNaturalist observations of lizards during or after interacting with cats in the Southern California study area. (A) Cat attacking a Southern Alligator Lizard (*Elgaria multicarinata*) that has autotomized its formerly complete, original tail; iNaturalist 2734344 by Annie Stevens; [www.inaturalist.org/observations/2734344](http://www.inaturalist.org/observations/2734344). (B) Cat (note head and ears in lower left of photo) attacking a Southern Alligator Lizard that has autotomized its tail; iNaturalist 6012370 by Dawn S. Chianese; [www.inaturalist.org/observations/6012370](http://www.inaturalist.org/observations/6012370). (C, D) Southern Alligator Lizard during and after an attack by a cat; iNaturalist 1733118 by Kristin Papoi and Violet Gibbs; [www.inaturalist.org/observations/1733118](http://www.inaturalist.org/observations/1733118). (E) Southern Alligator Lizard with a tail injury after being caught by a cat; the fresh tissue at the tail tip is a re-growing tail following an autotomy event several weeks prior to this observation; iNaturalist 1447779 by iNaturalist user biesman; [www.inaturalist.org/observations/1447779](http://www.inaturalist.org/observations/1447779). (F) Autotomized tail from a Southern Alligator Lizard on a neighborhood sidewalk following an attack by a cat; iNaturalist 2766323 by Patricia Simpson; [www.inaturalist.org/observations/2766323](http://www.inaturalist.org/observations/2766323). (G) Western Fence Lizard (*Sceloporus occidentalis*) caught by a cat; iNaturalist 23869292 by Sharon Nakata; [www.inaturalist.org/observations/23869292](http://www.inaturalist.org/observations/23869292). (H) Side-blotched Lizard (*Uta stansburiana*) caught by a cat; iNaturalist 8274946 by Maiz Connolly, who noted that "as soon as the cat dropped the lizard, the lizard dropped its tail, which wriggled enough to keep the cat's attention while it made its escape”. [www.inaturalist.org/observations/8274946](http://www.inaturalist.org/observations/8274946)


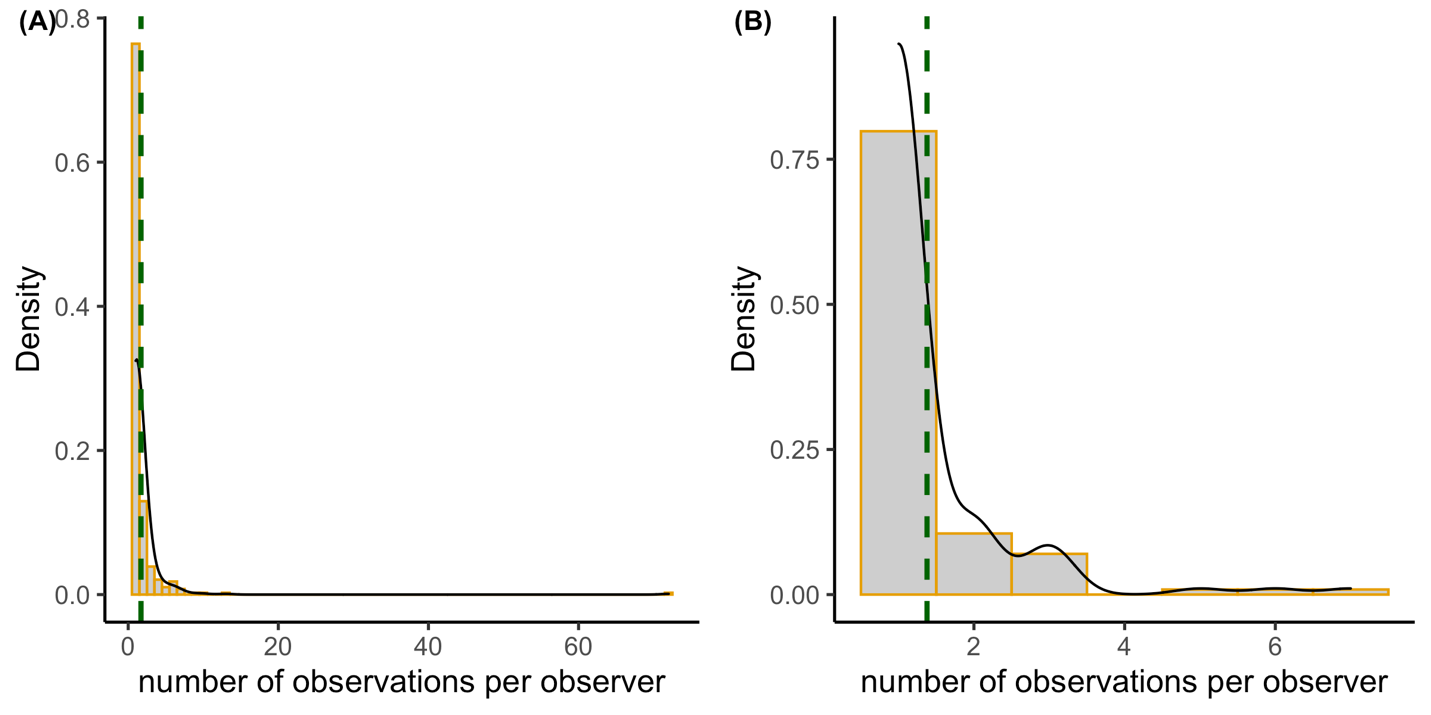


**Supplementary Fig. 2.** Histograms showing density of number of observations per observer in the (A) predation dataset (N = 723) and (B) parasitism dataset (N = 157). The dashed green line represents the mean number of observations per observer. Graphs were created in R (version 4.0.3; R Core Team 2020; [www.r-project.org](http://www.r-project.org)) using the *tidyverse* (Wickham et al. 2019; [CRAN.R-project.org/package=tidyverse](https://cran.r-project.org/package=tidyverse)) and *cowplot* (Wilke 2020; [CRAN.R-project.org/package=cowplot](https://cran.r-project.org/package=cowplot)) packages.


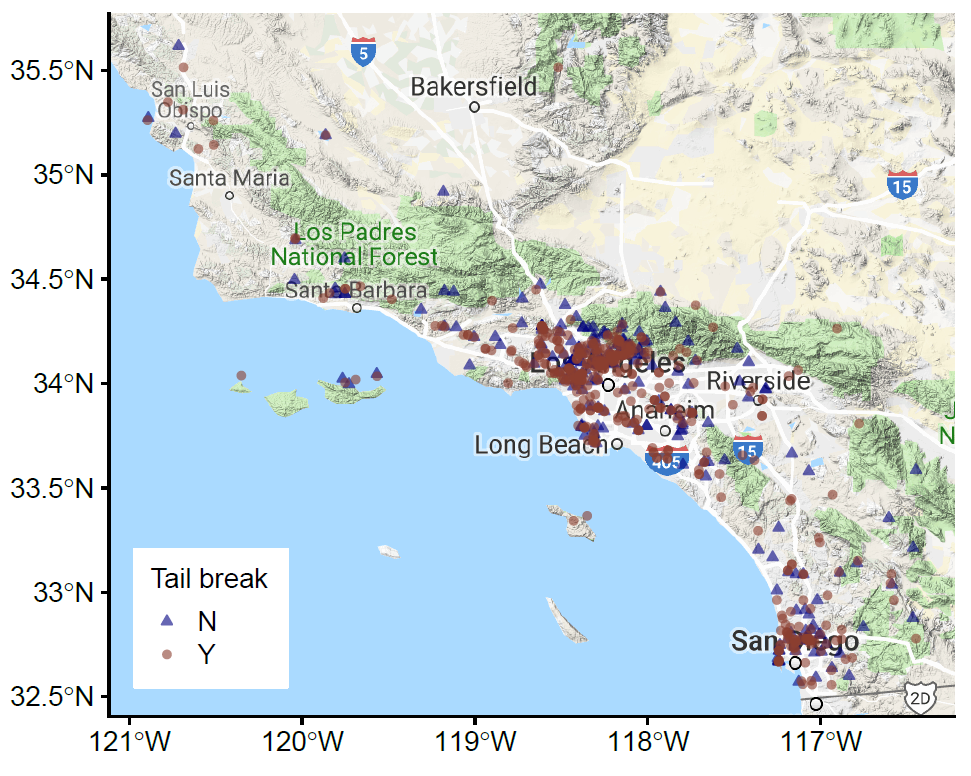


**Supplementary Fig. 3.** Map showing the geographic range of observations used in the predation portion of the study. Map was created in R (version 4.0.2; R Core Team 2020; [www.r-project.org](http://www.r-project.org)) using the *ggmap* (Kahle and Wickham 2013; [CRAN.R-project.org/package=ggmap](https://cran.r-project.org/package=ggmap)), *sf* (Pebesma 2018; [CRAN.R-project.org/package=sf](https://cran.r-project.org/package=sf) ), and *tidyverse* (Wickham et al. 2019; [CRAN.R-project.org/package=tidyverse](https://cran.r-project.org/package=tidyverse)) packages.

**Supplementary Table 1.** Data associated with Southern Alligator Lizards that had tick infections.

| Age | Sex | Imperviousness (%) | Number of ticks |
| --- | --- | --- | --- |
| Adult | Unknown | 0.00 | 2 |
| Adult | Unknown | 0.00 | 5 |
| Adult | Unknown | 0.00 | 1 |
| Adult | Unknown | 0.00 | 1 |
| Adult | Unknown | 0.00 | 1 |
| Adult | Unknown | 0.00 | 1 |
| Adult | Unknown | 0.03 | 5 |
| Adult | Unknown | 0.29 | 5 |
| Adult | Unknown | 0.47 | 5 |
| Adult | Unknown | 0.62 | 3 |
| Adult | Unknown | 1.05 | 3 |
| Adult | Unknown | 2.97 | 2 |
| Adult | Unknown | 4.72 | 1 |
| Adult | Unknown | 6.32 | 3 |
| Adult | Unknown | 8.14 | 3 |
| Adult | Unknown | 8.41 | 1 |
| Adult | Unknown | 25.09 | 2 |
| Adult | Unknown | 54.80 | 2 |
| Adult | Unknown | 63.77 | 1 |
| Adult | Male | 83.31 | 1 |
